# Digital technology: the next frontier for neonatal neuroprotection in the Americas

**DOI:** 10.1016/j.lana.2025.101304

**Published:** 2025-11-14

**Authors:** The Lancet Regional Health – Americas

For decades, the global newborn health agenda has been defined by a singular, urgent goal: survival among critically ill neonates. Thanks to relentless efforts, neonatal mortality rates have fallen worldwide, changing the focus now to the enhancement of long-term neurodevelopment and quality of life. In this context, neurological sequelae underlie much of the long-term burden among survivors of neonatal intensive care.

A systematic review published in 2025 analysed data from nearly 73,000 children in 47 studies across 12 countries, including data from Brazil and Ecuador. The study found that about 16% of preterm children in low-income and middle-income countries experience neurodevelopmental impairment, 5% have cerebral palsy, and 8–13% are affected by developmental delays. In this context, how can digital technologies bridge gaps in care and help protect the developing brains of the most vulnerable newborns?

In this issue, Variane and colleagues report a landmark achievement: the establishment of Brazil's multicentre digital neurocritical network, which remotely monitored brain function in more than 11,000 neonates across 79 hospitals. This nationwide digital health strategy integrated vital signs monitoring, real-time video amplitude-integrated and raw electroencephalography, and near-infrared spectroscopy, all fully supported by neonatologists and paediatric neurologists. Between July, 2017, and June, 2024, over 700,000 h of neuromonitoring and nearly 125,000 clinical interactions between remote monitoring hubs and bedside clinical teams were included in the study. To achieve what is documented as the largest implementation of digital neurocritical care in a middle-income country, extensive in-person and online training to use the technologies was delivered to multidisciplinary teams, strengthening local expertise, while a mixed-reality tool was piloted to support remote consultations and standardise neurological assessments. Continuous remote neuromonitoring, combined with digital education and standardised protocols, enabled earlier detection and intervention for neurological issues while reducing specialist shortages and care variability for high-risk infants.

Despite the promise of digital neurocritical care, its widespread implementation faces significant challenges. Reliable infrastructure is needed to support continuous, real-time monitoring; yet, in many regions, even basic electricity provision is unreliable and internet connectivity is far from stable. Additionally, robust regulatory frameworks must safeguard patient safety, data privacy, and legal compliance and sustainable financing models are essential to cover equipment, training, and ongoing operations.

Smaller, simpler solutions have been tested and proven valuable. For example, teleneonatology enables remote neonatologists to support hospitals via real-time telemedicine for critically ill newborns. A pilot study evaluated real-time video communication between neonatal intensive care units (NICUs) over 12 months. The programme facilitated 99 consultations for 50 families, showing high usability and satisfaction among both parents and health-care providers, speeding up medical decisions, enhanced care quality, and sometimes preventing unnecessary transfers. Furthermore, an economic modelling study evaluation found that, for neonates needing consultation in hospitals without NICUs, teleneonatology is significantly less costly than usual care, saving about US$11,000 per patient, primarily by reducing unnecessary transfers, according to analyses conducted within the US health-care system. By integrating digital neuromonitoring with teleneonatology, health-care systems could extend specialised neurological assessment and management to resource-limited settings, supporting early detection and intervention.

As digital technologies such as remote neuromonitoring and teleneonatology become integral to neonatal care, clinicians are faced with an unprecedented volume of complex data. Artificial intelligence (AI) offers a significant promise to transform neonatal care by helping clinicians interpret the large volume of complex, multimodal data generated by advanced neuromonitoring technologies. By integrating and analysing signals from electroencephalogram (EEG), vital signs, imaging, and other digital sources, AI could support accurate diagnosis and personalised treatment. For example, a multicentre randomised trial found that ANSeR, a machine-learning algorithm for neonatal seizure detection, improved the identification of seizure hours on EEG compared to conventional monitoring alone. The results suggest that it is safe and might be especially helpful in centres with little experience in EEG interpretation or restricted access to neurophysiology expertise. However, realising this potential will require addressing challenges related to testability, usability, bias, and transparency. Close collaboration among clinicians, data scientists, and policymakers is essential to ensure that AI tools are developed and implemented in a safe, equitable, and effective manner.

The impact of digital neurocritical care, including neuromonitoring, teleneonatology, data analytics, and AI, requires a multi-centre learning health system, rigorous outcome tracking, and a robust implementation strategy to scale up and provide access. Brazil has demonstrated how to build this capacity and infrastructure through the network managed by Protecting Brains & Saving Futures. Policymakers, funders, and professional societies must support national programmes with long-term goals to advance neuroprotection and development for newborns. The scientific community also plays a vital role in ensuring ethically conducted research and disseminating advances that drive the field forward. Our journal is committed to pioneering research in digital medicine as applied to neonatal care, and we encourage submissions that help shape this emerging and impactful field of applied digital medicine and critical care.

